# Complete mitochondrial genome and phylogenetic analysis of *Cyathostomum tetracanthum* (Rhabditida: Cyathostominae)

**DOI:** 10.1080/23802359.2020.1715289

**Published:** 2020-01-20

**Authors:** Shuanghui Wang, Peng Li, Chenguang Sun, Wei Liu, Yanzhen Bu

**Affiliations:** a Key Laboratory of Environment Change and Resources Use in Beibu Gulf, Nanning Normal University, Nanning, China;; b College of Life Sciences, Henan Normal University, Xinxiang, China

**Keywords:** Mitochondrial genome, *Cyathostomum tetracanthum*, phylogeny

## Abstract

The complete mitochondrial genome sequence of *Cyathostomum tetracanthum* was sequenced in the present study. It was determined to be 13,839 bp bases. The overall base composition was 30.93% A, 45.75% T, 6.97% C, and 16.35% G, with a very strong A + T bias (76.65%). The nucleotide sequence data of 12 protein-coding genes of *C. tetracanthum* and other 16 Strongylidae species were used for phylogenetic analyses. *Cyathostomum tetracanthum* was closely related with *Cylicocyclus* species rather than other *Cyathostomum* species. The complete mitogenome will facilitate taxonomy and systematics studies of Cyathostominae nematodes.

Strongylidae nematodes, which belongs to Rhabditida, can be further grouped into two subfamilies: Strongylinae and Cyathostominae (Lichtenfels et al. 2002). They inhabit the large intestine with a high prevalence in *Equus* species, which causes a series of clinical symptoms such as anemia, weight loss, and even death. *Cyathostomum tetracanthum*, which belongs to subfamily Cyathostominae, is a significant Strongylidae nematode. In the present study, the complete mitochondrial genome of *C. tetracanthum* was generated. Then, phylogenetic analysis of Strongylidae was performed using protein-coding genes in the mitochondrial genome.

Specimen of *C. tetracanthum* were isolated from the large intestine of infected donkey in Yanjin, Henan Province, China (114°19′E, 33°14′N). The voucher specimen was preserved in 95% ethanol and deposited in the Biological Specimen Museum of Henan Normal University, with an accession number YJ0068. The genomic DNA was extracted from a single specimen using standard phenol/chloroform methods (Sambrook et al. 1989). The DNA paired-end library was constructed and sequenced using Illumina Hiseq 4000 platform, then complete mitochondrial genome was assembled with SOAPdenovo v2.04 (Luo et al. 2012) and MITObim v1.6 (Christoph et al. 2013). The assembled genome was annotated using the DOGMA (Wyman et al. 2004) and then submitted into the GenBank database with accession number MN792800.

The complete mitochondrial genome of *C. tetracanthum* was 13,839 bp in length, which consists of 12 protein-coding genes (PCGs), 22 tRNA genes, two rRNA genes, and non-coding regions (NCR), all genes are encoded by the same strand. The overall base composition was 30.93% A, 45.75% T, 6.97% C, and 16.35% G, resulting in a very strong A + T bias (76.65%), in particular, the AT-rich region (84.34%). The size of the 22 tRNAs ranging from 44 to 63 bp. The rrnL (973 bp) is located between trnH and nad3, whereas the rrnS (696 bp) is located between trnE and trnS (UCN).

The phylogenetic position of *C. tetracanthum* was estimated using the Bayesian inference (BI), implemented in MrBayes version 3.1.2 (Ronquist and Huelsenbeck 2003). The best-fitting model (GTR + I + G) of sequence evolution for Bayesian analyses was obtained by Modeltest 3.7 (Posada and Crandall 1998) under the Akaike Information Criterion. Mitogenome sequences of 16 other Strongylidae species and *Bunostomum phlebotomum* were retrieved from GenBank. Analyses were performed using 12 protein-coding genes, using *B. phlebotomum* as outgroup.

Phylogenetic analysis showed that *C. tetracanthum* was closely related to *Cylicocyclus* species rather than other *Cyathostomum* species. Strongylidae nematodes grouped into two major clades: *Triodontophorus* species and others (Figure 1). Species of genus *Triodontophorus* clustered together with Cyathostominae species although they belonged to subfamily strongylinae, which was consistent with previous studies (Gao et al. 2017; Li et al. 2019). The mitogenome sequences will facilitate taxonomy and systematics studies of Cyathostominae nematodes in the future.

**Figure 1.**
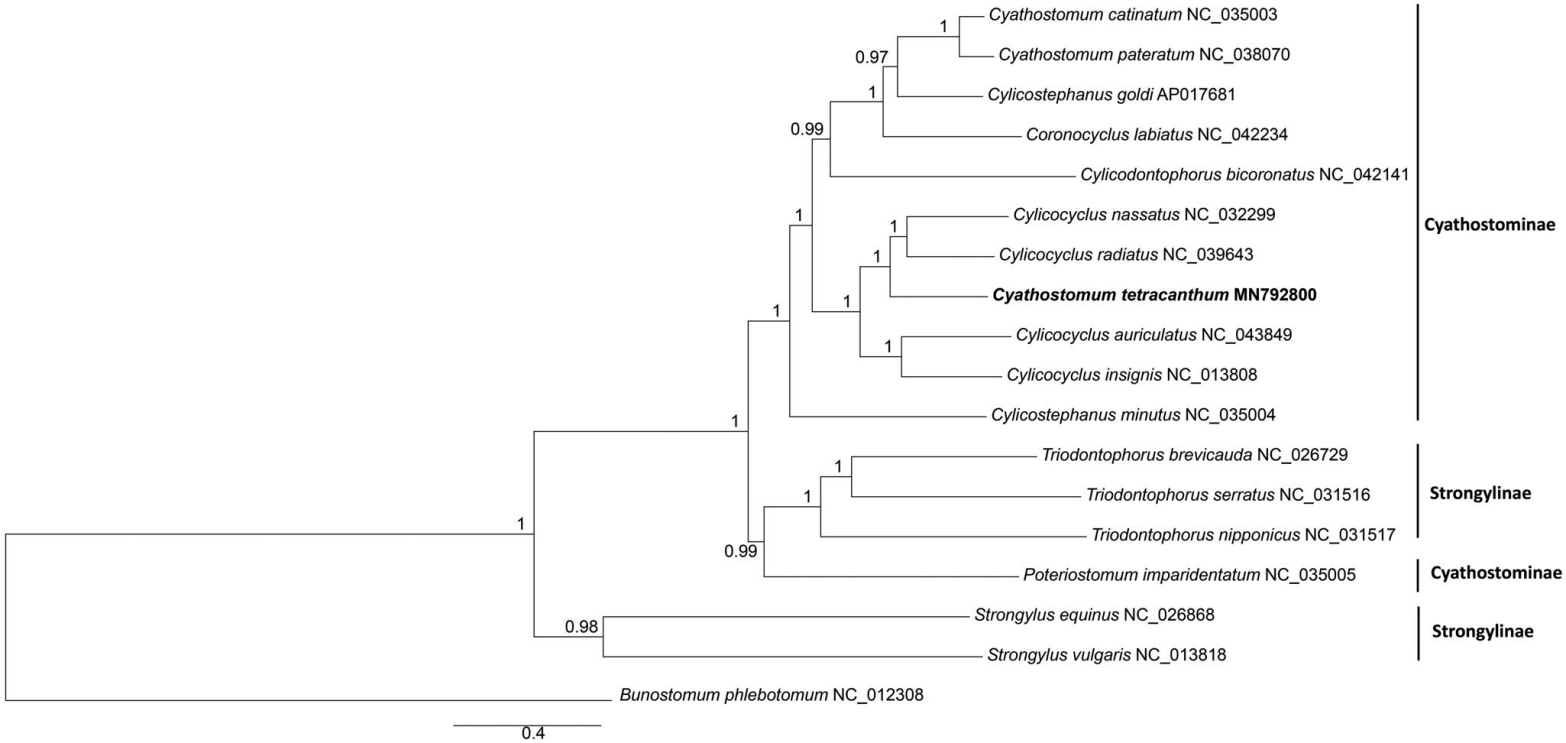
Bayesian 50% majority-rule consensus phylogenetic tree of *Cyathostomum tetracanthum* and other 16 species Strongylidae nematodes based on 12 protein-coding genes*. Bunostomum phlebotomum* were used as outgroups.
